# Antimicrobial Photodynamic Therapy Mediated by Curcumin-Loaded Polymeric Nanoparticles in a Murine Model of Oral Candidiasis

**DOI:** 10.3390/molecules23082075

**Published:** 2018-08-19

**Authors:** Vinicius Tatsuyuji Sakima, Paula Aboud Barbugli, Paulo Sérgio Cerri, Marlus Chorilli, Juliana Cabrini Carmello, Ana Cláudia Pavarina, Ewerton Garcia de Oliveira Mima

**Affiliations:** 1 School of Dentistry, São Paulo State University (UNESP), Araraquara 14801-903, São Paulo, Brazil; sakima888@gmail.com (V.T.S.); pabfarma@yahoo.com.br (P.A.B.); pcerri@foar.unesp.br (P.S.C.); cabrini.juliana@gmail.com (J.C.C.); pavarina@foar.unesp.br (A.C.P.); 2 School of Pharmaceutical Sciences, São Paulo State University (UNESP), Araraquara 14800-903, São Paulo, Brazil; chorilli@fcfar.unesp.br

**Keywords:** photochemotherapy, nanoparticles, keratins, mice, *Candida albicans*, oral candidiasis, curcumin

## Abstract

Antimicrobial photodynamic therapy (aPDT) has been proposed as an alternative method for oral candidiasis (OC), while nanocarriers have been used to improve the water solubility of curcumin (CUR). The aim of this study is to encapsulate CUR in polymeric nanoparticles (NPs) and to evaluate its photodynamic effects on a murine model of OC. Anionic and cationic CUR-NP is synthesized using poly-lactic acid and dextran sulfate and then characterized. Female mice are immunosuppressed and inoculated with *Candida albicans* (Ca) to induce OC. aPDT is performed by applying CUR-NP or free CUR on the dorsum of the tongue, followed by blue light irradiation for five consecutive days. Nystatin is used as positive control. Afterward, Ca are recovered and cultivated. Animals are euthanized for histological, immunohistochemical, and DNA damage evaluation. Encapsulation in NP improves the water solubility of CUR. Nystatin shows the highest reduction of Ca, followed by aPDT mediated by free CUR, which results in immunolabelling of cytokeratins closer to those observed for healthy animals. Anionic CUR-NP does not show antifungal effect, and cationic CUR-NP reduces Ca even in the absence of light. DNA damage is associated with Ca infection. Consecutive aPDT application is a safe treatment for OC.

## 1. Introduction

*Candida albicans* is an opportunist pathogen found in human mucosa that, under local or systemic conditions, might invade tissues and promote infection. *C. albicans* is the most prevalent species found in oral candidiasis (OC) [1]. OC is a common condition found in immunocompromised patients, especially those infected by human immunodeficiency virus (HIV), but also subjects under treatment with immunosuppressant agents, such as transplanted patients and those under chemotherapy [2]. Regarding those patients, the infection might spread from its original site and reach the bloodstream, causing a systemic infection known as candidaemia. Candidaemia is the fourth nosocomial infection with a high mortality rate—up to 50% [3].

OC is characterized by invasion of the hosts mucosal epithelium by the filamentous form (hyphae) of *C. albicans* [4]. Invasion occurs via two different ways: endocytosis, a process mediated by epithelial cells; or active penetration, in which a viable hypha penetrates through or between the epithelial cells. Regardless of the mechanism, invasion results in damage of the epithelium via necrosis and/or apoptosis [5]. To resist the external mechanical stress, the cytoplasm of epithelial cells has filaments of keratins called cytokeratins (CKs). CKs are protein constituents of the epithelial cytoskeleton found in the whole oral cavity and their specific level of expression is used to diagnose oral pathologies such as carcinomas [6]. While the CK13 is distributed in suprabasal layers of the stratified epithelium, the CK14 is expressed in non-differentiated basal cells of the stratified squamous epithelium. Both cytokeratins are expressed during morphogenesis of filiform and circumvallate papillae of rats [7,8]. However, to the best of the authors’ knowledge, the expression of CKs in *C. albicans* infection is still unknown.

The conventional antifungal agents used for local and systemic candidiasis have shown some drawbacks, with the development of resistant strains being a serious health concern [9]. Therefore, new therapies have been investigated, such as antimicrobial photodynamic therapy (aPDT), which combines a photosensitizer agent (PS) and a light source. The interaction between PS and light in the presence of oxygen produces reactive species, such as singlet oxygen and free radicals that promote cell damage and death [10,11]. aPDT has been extensively used against cancer [12] and some studies have demonstrated its potential in the treatment of infections [13], including those of the oral cavity [11].

Curcumin (diferuloymethane, CUR) is a natural compound found in turmeric with several therapeutic properties such as anti-inflammatory, antioxidant, anticancer, and antimicrobial activity [14]. Previous studies demonstrated that, for an antifungal effect, CUR requires lower concentrations (7.4 mg/L, equivalent to 20 μM) when used as PS in aPDT [15,16] than when used alone without light (minimum inhibitory concentration of 64 mg/L or 173.73 μM) [17]. Associated with a blue light source (440–485 nm), CUR promotes complete photoinactivation of planktonic cultures and 87.22% reduction in the metabolic activity of biofilm cultures of *C. albicans* [15]. When clinical isolates of *Candida* spp. are evaluated, CUR-mediated aPDT results in complete photoinactivation of planktonic cultures of *C. albicans* and *Candida tropicalis* and significant reduction of *Candida glabrata*, while biofilms show more than a 73% reduction in the metabolic activity and more than 52% in the total biomass [16]. Confocal laser scanning microscopy shows photosensitization of *C. albicans* biofilm only in the outmost layers of the biofilm [18]. When a murine model of oral candidiasis is evaluated, a single application of aPDT with CUR results in reduction of over 4.00 log_10_ of yeast from the tongue of mice, without histological features of fungal infection [19]. To extend the period of oral candidiasis and to evaluate a therapeutic protocol of consecutive aPDT applications in mice, Carmello et al., 2016 [20] performed four immunosuppression and five aPDT applications with photodithazine (PDZ) and light-emitting diode (LED) light on the tongues of mice. They observed a significant reduction of 3.00 log_10_ in the viability of *C. albicans* after aPDT compared with the control (untreated animals), as well as a significant increase in the expression of the tumor necrosis factor α (TNF-α) [20].

CUR also improves the wound healing process acting in several pathways. It inhibits the production of pro-inflammatory cytokines; stimulates healing growth factors; increases antioxidant enzymes, fibroblast numbers, granulation tissue formation, and collagen deposition; and accelerates neovascularization, re-epithelialisation, and wound closure [21,22,23,24]. However, the wound healing process has usually been evaluated by topical application of CUR in cutaneous [25] and burn wounds [26], and few studies have evaluated infected tissues [27,28].

Despite these therapeutic effects, clinical use of CUR is limited because of insolubility in water (hydrophobicity), poor stability (hydrolytic, proteolytic, and photodegradation), and low bioavailability. Therefore, drug delivery systems have been investigated to overcome these drawbacks [24,29,30]. Research has been carried out to find more efficient drug delivery systems, associating CUR with different polymers forming a stable nano-structured polymeric system in this context. Nanoformulations of CUR have been used in infected tissues of rodents to evaluate its antimicrobial and healing efficacy. An ethosomal formulation of CUR inhibited the growth of *Pseudomonas aeruginosa* and improved the healing of burn wounds of rats [27]. CUR in nanoparticles (NP) of chitosan and polyethylene glycol reduced the viability of planktonic cultures of methicillin-resistant *Staphylococcus aureus* (MRSA) and *P. aeruginosa* in vitro, as well as that of MRSA in burn wounds of mice, which showed a faster wound closure, better maturation of granulation tissue, and improved neovascularization and collagen maturation after topical application [28].

A recent study synthesized CUR in nanoparticles (CUR-NPs) of poly-lactic acid (PLA) and dextran (DEX) and evaluated its antimicrobial efficacy in vitro. CUR-NP showed light absorbance and photodegradation similar to free CUR, and its photodynamic antimicrobial activity against planktonic cultures of *C. albicans*, *Streptococcus mutans*, and MRSA was higher compared with the results of biofilms of these species [31]. The synthesized CUR-NP is used in a murine model of oral candidiasis to evaluate its photodynamic effect in the present investigation. Moreover, the detection of epithelial CKs and the DNA damage in the host’s tissue also are evaluated.

## 2. Results

### 2.1. Synthesis and Characterization of CUR-NPs

#### 2.1.1. Physicochemical Properties and Morphology Analysis

**Figure 1:**
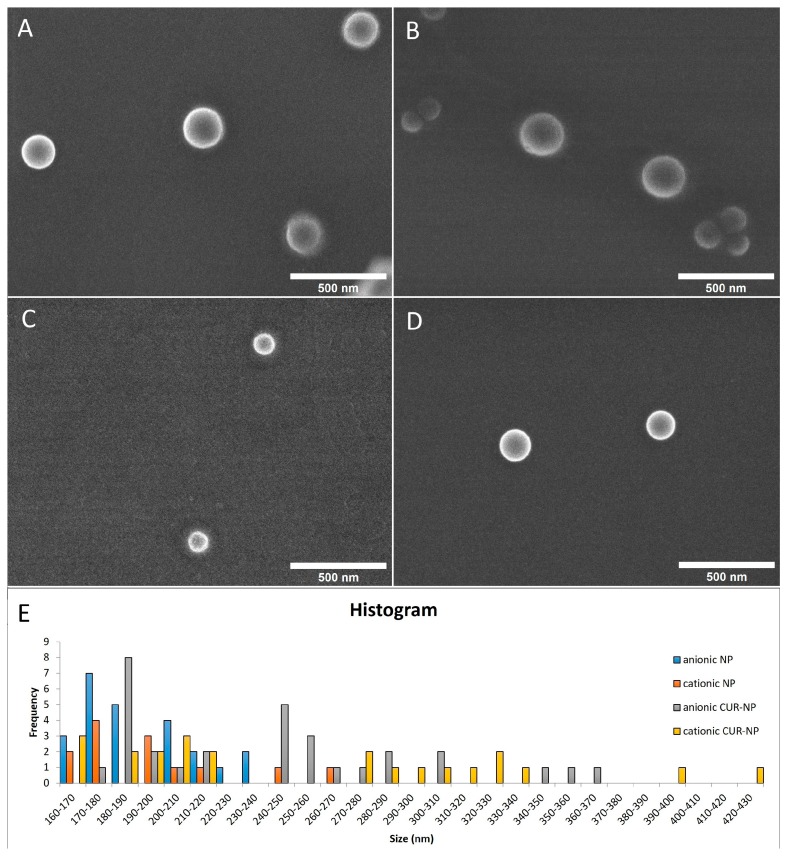
FEG-SEM of NP formulations with and without CUR: (**A**) anionic CUR-NP (magnification: ×60,000); (**B**) cationic CUR-NP (magnification: ×60,000); (**C**) anionic NP without CUR (magnification: ×60,000); (**D**) cationic NP without CUR (magnification: ×60,000); and (**E**) histogram showing size of all formulations.

The highest CUR concentration encapsulated in NPs was 260 μM, as higher concentrations resulted in aggregation of CUR. Anionic and cationic CUR-NPs were synthesized, and anionic and cationic NPs without CUR were synthesized as controls. The physicochemical properties were evaluated within a period interval of 0–29 days after the synthesis (Table 1). Images of field emission gun scanning electron microscopy (FEG-SEM) (Figure 1A–D) were captured nine days after the synthesis, showing spherical nanoparticles.

#### 2.1.2. Absorption Spectrum and Photostability

**Figure 2:**
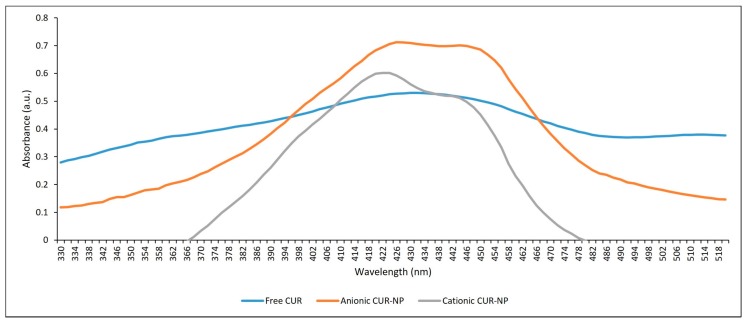
Absorption spectrum of free CUR, anionic and cationic CUR-NP.

During the light absorption study, the absorbance signals of free CUR, and anionic and cationic CUR-NP were similar in the blue range of the UV-visible spectrum. The free CUR presented maximum absorbance at 430 nm and both anionic and cationic CUR-NP at 426 nm and 424 nm, respectively (Figure 2). Although the wavelength values were close, the absorbance of the free CUR formulation was lower than those of the CUR-NPs, with values of 0.53, 0.712, and 0.602 a.u. observed for free CUR, and anionic and cationic CUR-NP, respectively.

**Figure 3:**
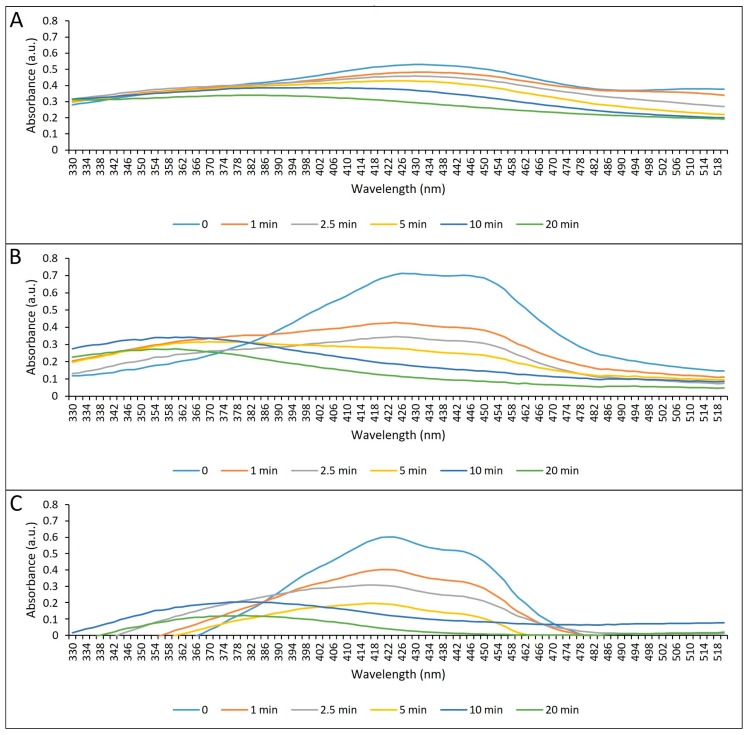
Photodegradation of free CUR, and anionic and cationic CUR-NPs after different periods of illumination with blue LED. (**A**) Free CUR; (**B**) anionic CUR-NP; (**C**) cationic CUR-NP.

During the photostability study, the authors observed a reduction of the maximum absorption signal of the formulations proportional to the illumination time for all the formulations. The free CUR showed a degradation of 44.7% after illumination of 43.2 J/cm^2^, corresponding to 20 min. Concerning anionic and cationic CUR-NP, we observed degradation of 84.0% and 94.0%, respectively, after 20 min of illumination (Figure 3).

#### 2.1.3. Encapsulation Efficiency (EE) and Release of CUR from NPs

Regarding EE, values of 60.84% and 73.81% were observed for the anionic and cationic CUR-NPs, respectively.

**Figure 4:**
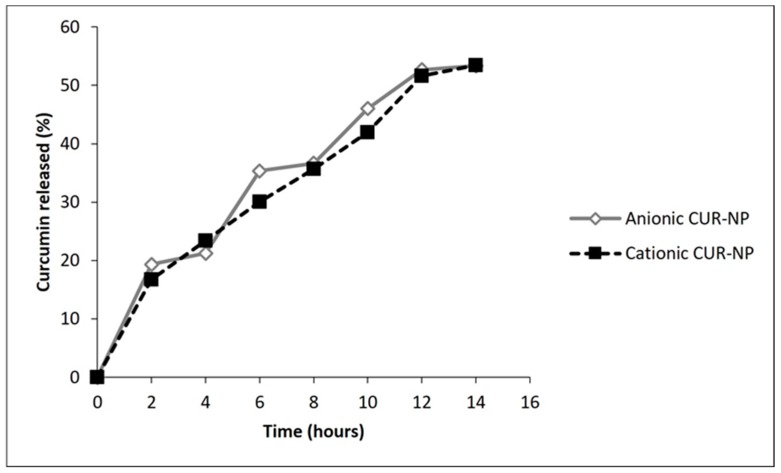
Release of CUR from anionic and cationic NPs (in percentage).

Concerning CUR release, a slow and progressive CUR release from NPs was observed. After 12 h, release values of 52.6% and 51.6% of CUR from the anionic and cationic NP, respectively, were observed. (Figure 4).

### 2.2. In Vivo Study

#### 2.2.1. Antifungal Treatments of Oral Candidiasis in Mice

Tongues of mice inoculated with yeast showed the mean value of 4.95 log_10_ of colony-forming units per mL (CFU/mL) of *C. albicans* (control group). However, white patches on the dorsum of the tongue were observed only for some animals, consequently, a macroscopic analysis of lesions was not performed. When free CUR at 80 μM was evaluated, no antifungal photodynamic effect was observed (data not shown). Therefore, free CUR at 260 μM was evaluated because this was the highest CUR concentration encapsulated in NPs. A total of 13 animals died before the end of the experiments (six died during *C. albicans* inoculation, and seven after being allocated in the groups of treatments). Each treatment was performed daily during five consecutive days.

**Figure 5:**
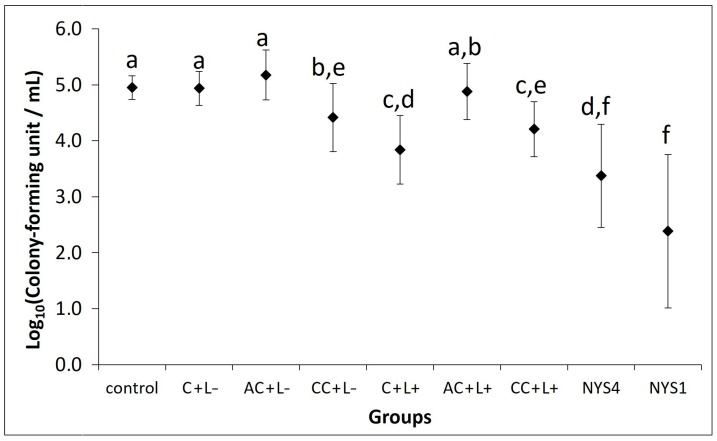
Mean values of log_10_ (CFU/mL) of *C. albicans* recovered from the tongue dorsum on the last day of treatment (*n* = 24). The control group corresponded to infected animals that did not receive any treatment (C − L −); C + L+, AC + L+, and CC + L+ groups: animals treated with free CUR, and anionic and cationic CUR-NP, respectively, associated with blue LED light; C + L−, AC + L–, and CC + L− groups: evaluation of each PS alone (free CUR, anionic and cationic CUR-NP, respectively); NYS1 and NYS4 groups: animals treated with Nystatin 100,000 international units (IU) once (under anesthesia) and four times daily, respectively. Different letters represent statistical differences between the groups (*p* < 0.05). Error bars: standard deviation.

The quantification of *C. albicans* recovered from tongues of mice after five days of treatment in all groups is depicted in Figure 5. aPDT mediated by free CUR (C + L+ group) resulted in a significant (*p* < 0.001) reduction of 1.11 log_10_ in the viability of *C. albicans* recovered from tongues of mice compared with the control group (C − L−). Mice treated only with free CUR (C + L− group) showed no significant difference compared with the control (*p* = 1.000). Regarding anionic CUR-NP, no significant difference in the log_10_ (CFU/mL) was observed (*p* ≥ 0.421) for animals submitted to photosensitizer (PS) only (AC + L−) or aPDT (AC + L+) compared with the control. Conversely, cationic CUR-NP promoted a significant reduction (*p* < 0.009) of 0.74 and 0.44 log_10_ in the presence of (CC + L+) and absence of light (CC + L−), respectively, compared with the control group. Although mice treated only with cationic CUR-NP (CC + L−) showed no significant difference (*p* = 0.928) to those treated with cationic CUR-NP-mediated aPDT (CC + L+), the former demonstrated a significant difference (*p* = 0.048) compared with aPDT mediated by free CUR (C + L+), which was similar (*p* = 0.360) to aPDT mediated by cationic CUR-NP (CC + L+).

Higher reductions were observed for mice treated with nystatin (NYS), as significant reductions (*p* < 0.001) of 1.58 and 2.57 log_10_ were obtained for NYS four times/day (NYS4) and NYS once a day under anesthesia (NYS1), respectively, compared with the control group. When all effective antifungal groups were compared, NYS4 and NYS1 demonstrated no significant difference between themselves (*p* = 0.109), but lower values (*p* ≤ 0.011) than those found in groups treated with cationic CUR-NP (CC + L− and CC + L+). Moreover, NYS1 showed the highest reduction of log_10_ (CFU/mL) with a significant difference (*p* = 0.001) compared with aPDT mediated by free CUR (C + L+), which was similar to NYS4 (*p* = 0.516).

#### 2.2.2. Histological Analysis—Periodic Acid-Schiff (PAS)

**Figure 6:**
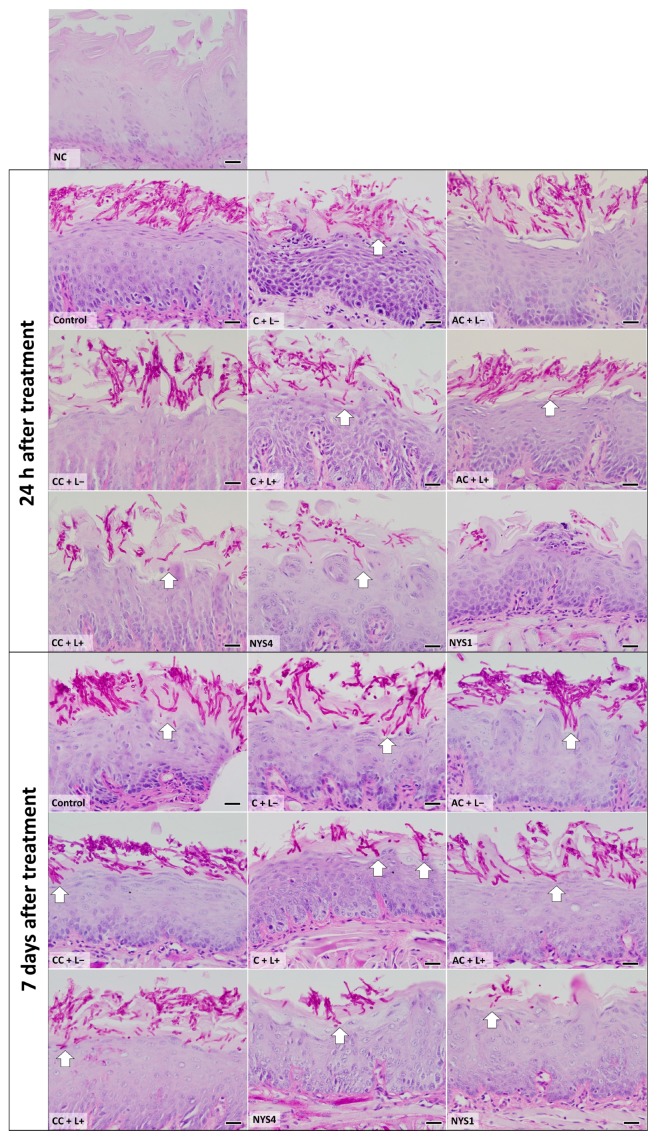
Light micrographs showing sections of tongue mucosa submitted to the periodic acid-Schiff (PAS) histochemical method. The light micrographs show the epithelium tongue of healthy, infected, and treated animals, euthanized 24 h and seven days after treatment. NC group: healthy and untreated animals; Control group: infected animals that did not receive any treatment (C − L−); C + L+, AC + L+, and CC + L+ groups corresponded to the animals treated with aPDT mediated by free CUR, anionic and cationic CUR-NP, respectively, and blue LED light; C + L−, AC + L−, and CC + L− groups corresponded to evaluation of each PS alone (free CUR, and anionic and cationic CUR-NP, respectively); NYS1 and NYS4 groups: animals treated with Nystatin 100,000 IU once (under anesthesia) and four times daily, respectively. Arrows: invasion of *C. albicans* in the subjacent epithelium. Magnification: ×40. Scale bar: 20 μm.

Histological images from tongues of mice from all groups are shown in Figure 6. The epithelium showed alteration of the filiform papillae in the keratin region, presence of the filamentous form of *C. albicans* in the epithelium stratum corneum with invasion in the stratum spinosum, and few inflammatory cells in the lamina propria of the lingual mucosa in all infected animals compared with the healthy animals (negative control, NC group). Mice treated with aPDT (C + L+) with free CUR and NYS4 also showed these histological alterations, but with fewer fungal cells compared with animals from the control (C − L−), PS only (free CUR, anionic and cationic CUR-NP), and aPDT groups with anionic and cationic CUR-NP, which showed similar histological features for both periods of observation (24 h and seven days after treatment). The group of animals NYS1 (euthanized 24 h and seven days after the treatment) exhibited the lowest number of fungal filaments in the epithelium tissue.

#### 2.2.3. Cytokeratins (CKs) 13 and 14 Immunolabelling

**Figure 7:**
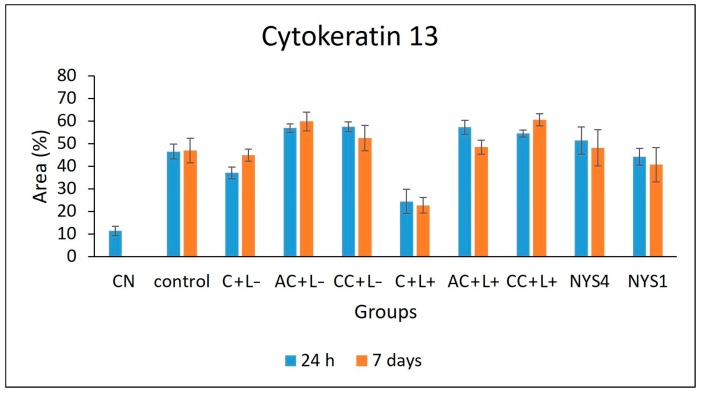
Mean values of CK13 immunolabelling area measured by ImageJ software (*n* = 2). NC group: healthy and untreated animals; control group: infected animals that did not receive any treatment (C − L−); C + L+, AC + L+, and CC + L+ groups: animals treated with aPDT mediated by free CUR, anionic and cationic CUR-NP, respectively, and blue LED light; C + L−, AC + L−, and CC + L− groups: evaluation of each PS alone (free CUR, and anionic and cationic CUR-NP, respectively); NYS1 and NYS4 groups: animals treated with Nystatin 100,000 IU once (under anesthesia) and four times daily, respectively. Error bars: standard deviation.

**Figure 8:**
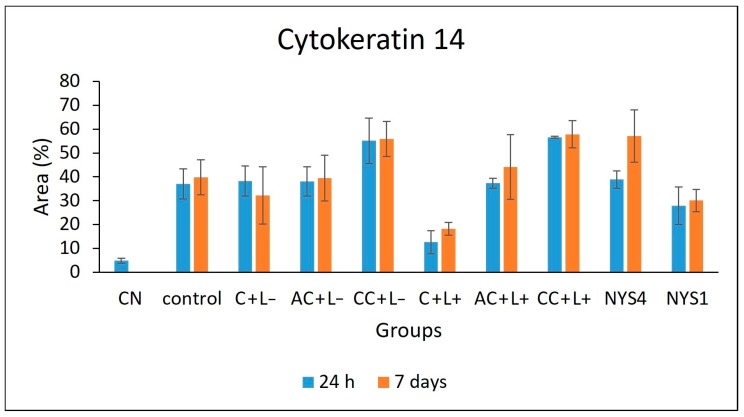
Mean values of CK14 immunolabelling area measured by ImageJ software (*n* = 2). NC group: healthy and untreated animals; control group: infected animals that did not receive any treatment (C − L−); C + L+, AC + L+, and CC + L+ groups: animals treated with aPDT mediated by free CUR, anionic and cationic CUR-NP, respectively, and blue LED light; C + L−, AC + L−, and CC + L− groups: evaluation of each PS alone (free CUR, and anionic and cationic CUR-NP, respectively); NYS1 and NYS4 groups: animals treated with Nystatin 100,000 IU once (under anesthesia) and four times daily, respectively. Error bars: standard deviation.

**Figure 9:**
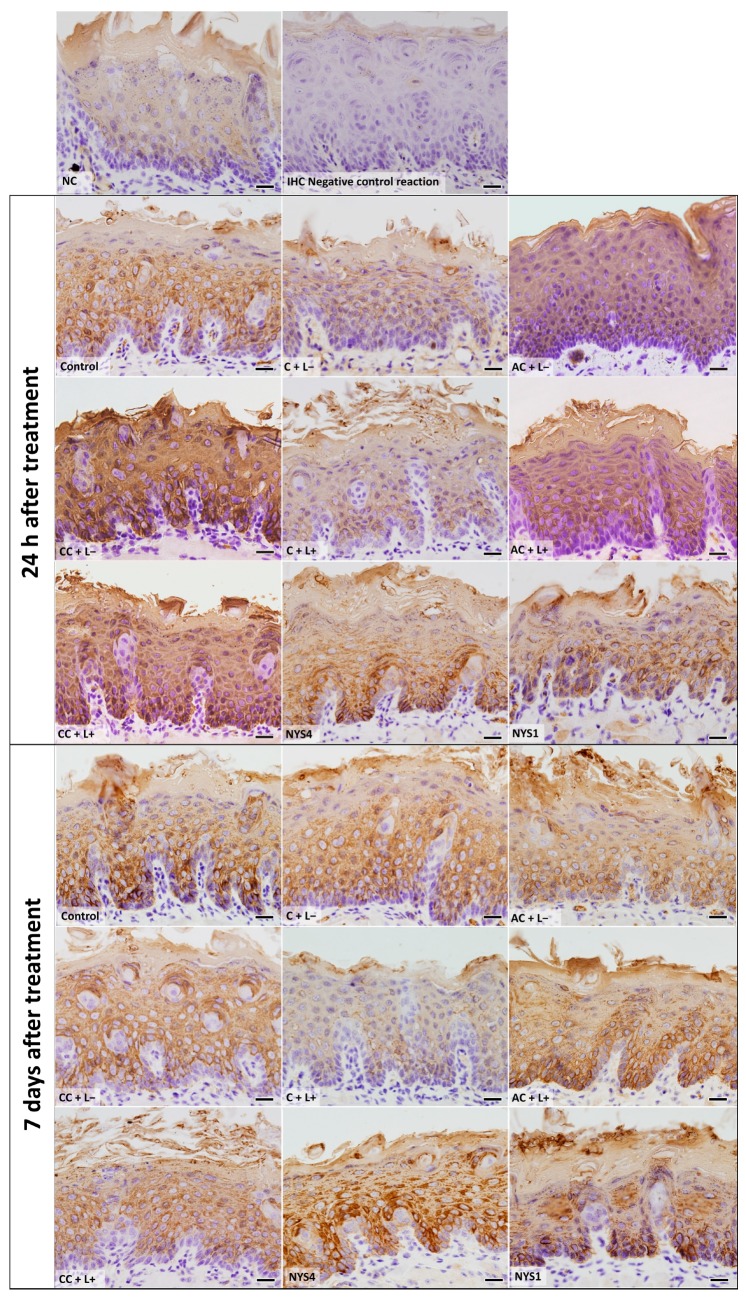
Light micrographs showing portions of sections of the tongue dorsal mucosa submitted to IHC for CK13 detection and counterstained with haematoxylin. NC group: healthy and untreated animals; Control group: infected animals that did not receive any treatment (C − L−); C + L+, AC + L+, and CC + L+ groups: animals treated with aPDT mediated by free CUR, anionic and cationic CUR-NP, respectively, and blue LED light; C + L−, AC + L−, and CC + L− groups: evaluation of each PS alone (free CUR, and anionic and cationic CUR-NP, respectively); NYS1 and NYS4 groups: animals treated with Nystatin 100,000 IU once (under anesthesia) and four times daily, respectively. Magnification: ×40. Scale bar: 20 μm.

**Figure 10:**
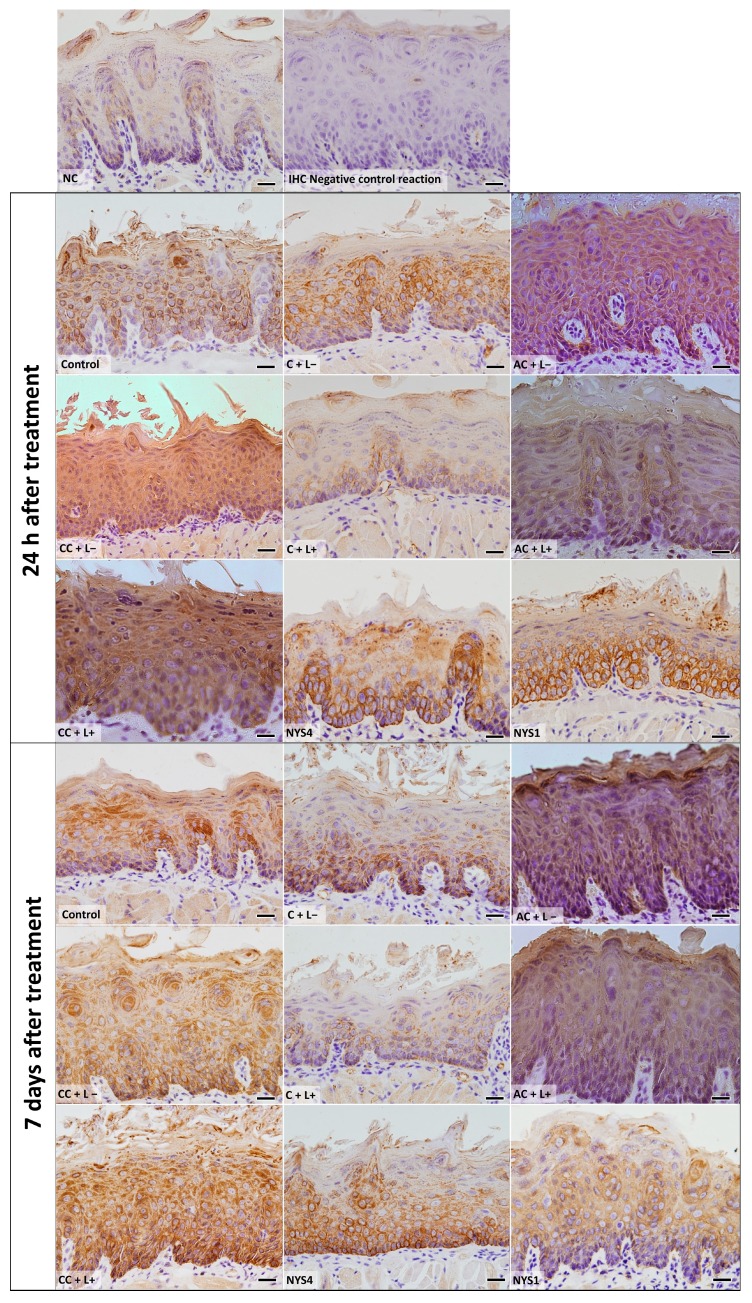
Light micrographs showing portions of sections of the tongue dorsal mucosa submitted to IHC for CK14 detection and counterstained with haematoxylin. NC group: healthy and untreated animals; Control group: infected animals that did not receive any treatment (C − L−); C + L+, AC + L+, and CC + L+ groups: animals treated with aPDT mediated by free CUR, anionic and cationic CUR-NP, respectively, and blue LED light; C + L−, AC + L−, and CC + L− groups: evaluation of each PS alone (free CUR, and anionic and cationic CUR-NP, respectively); NYS1 and NYS4 groups: animals treated with Nystatin 100,000 IU once (under anesthesia) and four times daily, respectively. Magnification: ×40. Scale bar: 20 μm.

The evaluation of CK13 and CK14 by immunohistochemistry (IHC) (Figure 7 and Figure 8) demonstrated different patterns in the immunolabelling according to the groups of treatments and periods. However, inferential statistics were not performed because only two animals from each group were evaluated (three sections for each animal). In healthy animals (NC), the lowest CK13 and CK14 immunolabelling was observed (only 5% and 11% of the epithelial area, respectively, was immunolabelled). Conversely, the non-treated infected mice (control group, C − L−) showed a more intense immuno-expression of both CKs at 24 h and seven days after treatment (46–47% for CK13, and 37–39% for CK14). Compared with the control group, a less intense immuno-expression for CK13 was observed in the animals treated with free CUR (C + L−) assessed after 24 h (37%) and NYS1 for both periods (40–44%), and the lowest immuno-expression among the infected animals was observed in the aPDT group with free CUR (C + L+) for both periods evaluated (22–24%, Figure 9). The highest CK13 immunolabelling was observed in groups treated with both CUR-NP (without and with light: AC + L−, CC + L−, AC + L+, CC + L+) and NYS4 (48–60%). A similar finding was observed for CK14 (Figure 10), as infected animals submitted to aPDT with free CUR showed the lowest immuno-expression (12–18%), followed by NYS1 (27–30%). The highest immunolabelling was verified in mice treated with cationic CUR-NP (without and with light, CC + L− and CC + L+, respectively) at both periods, and NYS4 after seven days (55–57%), while animals treated with anionic CUR-NP without and with light (AC + L− and AC + L+, respectively) for both periods and NYS4 evaluated after 24 h showed values (37–44%) similar to the control group.

#### 2.2.4. Terminal Deoxynucleotidyl Transferase (TdT)-Mediated Deoxyuridine Triphosphate (dUTP)-Biotin Nick End Labeling (TUNEL) Assay

**Figure 11:**
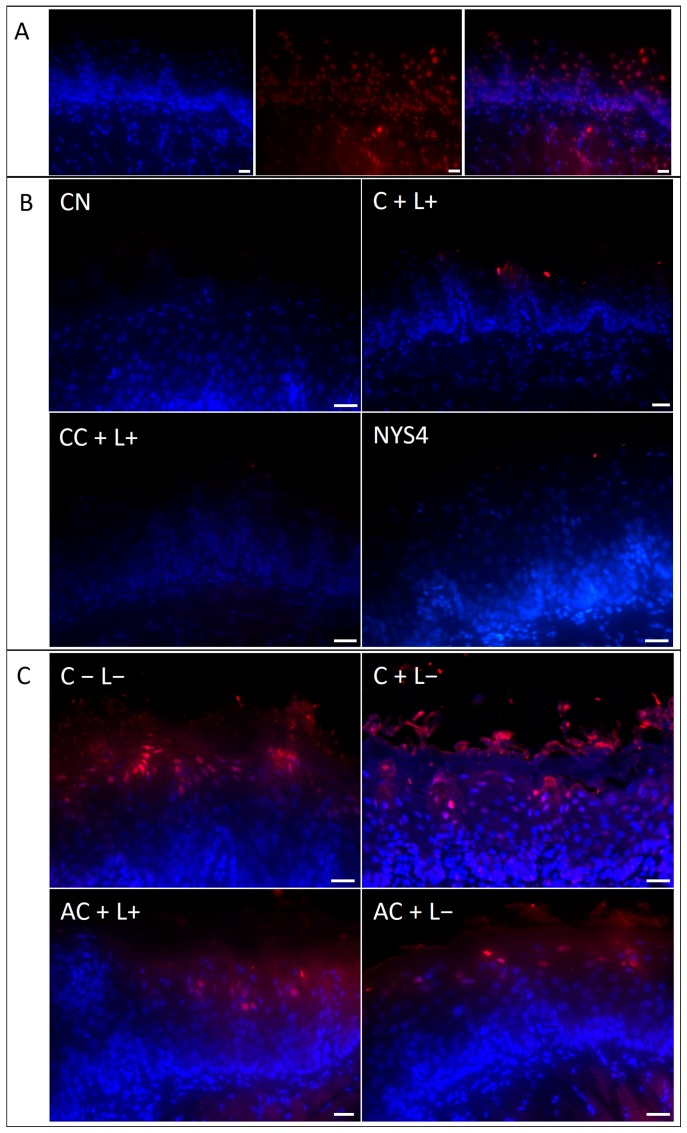
TUNEL assay of sections from the tongue of mice. (**A**) Positive control of the reaction (DNAse-treated sample) showing blue channel, red channel and overlay from left to right. (**B**) These four images show no/few cells with DNA damage (red fluorescence) on the tongue mucosa and represent animals killed at 24 h after treatments and mice from the groups NC, C + L+, CC + L−, CC + L+, NYS4, and NYS1 seven days after treatments. (**C**) High number of DNA-damaged cells (red fluorescence) of the animals from C − L−, C + L−, AC + L+, and AC + L− groups seven days after treatment. Scale bar: 20 μm.

Following 24 h of treatments, mice from the experimental groups showed no/few epithelial cells with DNA damage (red fluorescence) on the tongue mucosa (Figure 11B), as well as animals from NC, C + L+, NYS1, NYS4, CC + L−, and CC + L+ killed seven days after the treatments, similar to healthy mice (negative control, NC group). Increased number of DNA-damaged cells were observed on the tongue tissue of mice from C − L−, C + L−, AC + L−, and AC + L+ seven days after treatments (Figure 11C). The slide used as a positive control of the reaction showed many DNA-damaged cells in the tongue tissue, especially in the epithelial layers (Figure 11A).

## 3. Discussion

CUR-NPs synthesized by the nanoprecipitation [32] method using PLA and DEX showed similar properties to those previously described [31], demonstrating that the synthesis process used to obtain CUR-NP was reproducible. Although the synthesized formulations were stable in solution, their physicochemical features (size, PDI and Zeta potential) might change over time. Therefore, all experiments were performed immediately after the synthesis. Even 29 days after the synthesis, the values found in DLS were comparable to other studies [33,34,35,36,37], which employed different polymers and methods to synthesize CUR-NP. During the present investigation, the size of CUR-NPs was also evaluated by FEG-SEM, which demonstrated a smaller size than that found in DLS, due to the shrinkage of the NPs as a consequence of the drying procedure required for FEG-SEM [38].

The encapsulation process did not change the absorption spectra of CUR in this study, as the maximum absorbance values obtained from free CUR, and anionic and cationic CUR-NP were observed at blue wavelengths, similar to those reported in previous studies in which CUR showed absorbance between 400–470 nm [15,39]. Moreover, the photostability assay showed that all the PSs demonstrated photodegradation higher than 44.7% after 43.2 J/cm^2^ of blue light. This result agrees with a previous investigation [31], in which free CUR showed lower photodegradation compared with CUR-NP. Other studies found degradation values of 46–86% after 9.4 J/cm^2^ of light [39], and 70% after 0.4 J/cm^2^ of light [15].

In the present study, the percentage of EE was observed to be 60.84% for anionic CUR-NP and 73.81% for cationic CUR-NP. These values agree with other investigations that verified EE from 46.9% to 97% [33,37,40,41,42,43]. The release of CUR from NP was slow and progressive. After 12 h, approximately 52% of CUR was released from the NP. The authors’ previous study verified that 48 h was needed to release 96% of CUR from NP [31], and other investigations also observed a progressive release of CUR from 20% to 92.8% during periods from 6 h to 15 days [28,33,37,44,45].

During the present study, a treatment protocol of aPDT with free CUR topically applied on the dorsum of the tongue of mice with OC resulted in a reduction of 1.11 log_10_ in the viability of *C. albicans*, while aPDT mediated by anionic CUR-NP demonstrated no antifungal effect, and cationic CUR-NP reduced the fungal load (0.44–0.74 log_10_) even in the absence of light. These results agree with the authors’ previous in vitro study, in which the same PSs were used against biofilm of *C. albicans* and resulted in photoinactivation of 2.26 log_10_ for free CUR, no antifungal effect for anionic CUR-NP and reductions higher than 2.00 log_10_ for cationic CUR-NP even in the absence of light [31]. The inefficacy of anionic CUR-NP in inactivating the biofilm of *C. albicans* could be explained by the negative charge of the formulation that promotes repulsion against the negative charge of biofilm matrix [46] and the *C. albicans* cell wall [47]. Some studies have demonstrated that cationic PSs are more effective in aPDT compared with anionic PSs [48,49]. Hence, a cationic CUR-NP was also synthesized and evaluated in this study, and the antifungal effect observed even in the absence of light could be ascribed to the cetyltrimethylammonium bromide (CTAB) in the formulation, which, per se, has an antimicrobial effect [50]. This finding reinforces the authors’ previous in vitro study, which demonstrated that cationic CUR-NP was toxic to microbial and mammal cells, while anionic CUR-NP was non-toxic [31].

Another possible reason for the absence of an antifungal effect of anionic CUR-NP is the slow CUR release from NP; the aPDT protocol established for the pre-irradiation time of PS in the tongue of animals corresponded to 20 min. During the release test of CUR, less than 10% of CUR in the CUR-NP formulations was released in 20 min, which could have reduced the antimicrobial efficacy of aPDT. CUR in NP of chitosan and polyethylene glycol promoted a significant reduction in bacterial counts when applied topically for seven days on the burn wounds of mice infected with MRSA (more than 1.00 log_10_) [28]. During the present study, application of CUR-NP for five consecutive days would result in total release of CUR from NP. However, the topical application on the tongue does not keep the CUR-NPs in the mucosa because animals eliminate the formulation.

The results of the present investigation demonstrate that aPDT resulted in reduced photoinactivation of *C. albicans*. According to the American Society of Microbiology (ASM), an antimicrobial approach should promote reduction of at least 3.00 log_10_ [51]. A previous study demonstrated that the CUR-mediated aPDT promoted reduction of 4.00 log_10_ of *C. albicans* recovery from the tongues of mice with induced OC when a single topical application of aPDT was used [19]. The low reduction in the fungal viability after aPDT in the present research might be associated with the purity of CUR used (>65%). In another investigation, five aPDT applications with PDZ reduced the fungal viability in 3.00 log_10_ [20], and no death was observed. This higher reduction might be associated with the different PS used. Another reason for the higher reduction in the fungal viability in these studies might be the method of inoculum cultivation; while the present investigation used a suspension of *C. albicans* in the mid-log phase of growth, the other studies used an overnight culture. Conversely, in the present investigation, 13 mice died during the experiments, which was an unexpected result. The death of these mice might be attributed to the extended period of infection and the consecutive aPDT application, which required daily anesthesia of animals. A long period of OC might predispose animals to a systemic infection due to the immunosuppressive condition of mice and the ability of *C. albicans* to invade tissues, which was observed in the histological analysis. Moreover, after anesthesia, a recovery period for animals of longer than 24 h would be necessary before another aPDT application. Although the previous study [20] reported no death, no weight loss, and no behavior alteration, the possibility of systemic infection and death should be taken into consideration when OC is reproduced. The deaths observed in the present investigation suggest that impaired systemic health of mice would also reduce the efficacy of the treatments performed. Therefore, the impaired health of mice might also explain the reduced antifungal efficacy of aPDT in this study, because a competent immune system is also required to improve the host defense against *C. albicans*.

Treatment with nystatin four times a day was performed in order to simulate a clinical antifungal therapy, and it showed similar antifungal efficacy to aPDT. A clinical trial also demonstrated that topical application of nystatin showed similar results to aPDT for denture-associated OC [52]. Surprisingly, nystatin once a day in anesthetized animals was the most effective treatment in this study, resulting in 2.57 log_10_ of reduction. The highest reduction of fungal viability achieved by nystatin once a day might be justified by the anesthesia, which enables a longer period of action by the antifungal agent. Nystatin applied in non-anesthetized mice is removed from the mouth when mice feed, intake water, lick, and more. However, even the best result achieved with nystatin in the present investigation is below that recommended by ASM. Carmello et al., 2016 [20] also observed a high reduction of *C. albicans* (3.20 log_10_) after nystatin once a day in anesthetized mice. Although aPDT was performed in anesthetized animals, its antimicrobial action occurs only when the PS is activated by light, because the produced reactive oxygen species have a very short lifetime (microseconds) [11]. Therefore, as the reduction in the viability of *C. albicans* observed with aPDT once a day was similar to nystatin four times a day, the antifungal effect of aPDT might be considered relevant.

The histological analysis demonstrated that the amount of *C. albicans* on the keratin layer of the tongue corroborates with the CFU/mL results. Therefore, few filamentous cells were observed in the groups treated with nystatin and aPDT with free CUR. Few/scarce inflammatory cells were observed in all mice infected with *C. albicans*, suggesting that the treatments did not exacerbate the inflammatory response of tissues. These histological findings were similar for both periods of observation (24 h and seven days after the treatments), corroborating with a previous study [20]. However, Carmello et al, 2016 [20] did not observe fungal invasion in the epithelium of animals treated with nystatin, while in the present investigation, fungal invasion was also verified in animals treated with nystatin. Other in vivo studies evaluated the histopathological features of epidermal wound healing treated with CUR-NP. When the healing of burn wounds of mice was evaluated, CUR-NP reduced the inflammatory infiltrate, and improved the collagen deposition and the neovascularization of wounds [28]. CUR in NP of poly (lactic-co-glycolic acid) also improved the healing of excisional wounds of mice, which showed better re-epithelialization, and an increase in the collagen formation in the connective tissue [33]. A composite of hydrogel with CUR encapsulated in micelles also improved the wound healing of rats, which showed increased re-epithelialization, granulation tissue, fibroblast proliferation, and deposition of collagen [53]. CUR in pluronic gel also accelerates the wound healing of diabetic rats [25]. However, in these studies of wound healing, slow release of CUR from the delivery systems is a positive factor because CUR is maintained in the tissue during the wound-healing period. Conversely, for topical aPDT, the maximum of CUR should ideally be released from the nanocarrier during light activation to produce reactive oxygen species and, consequently, the antimicrobial effect.

As this study evaluated a superficial infection of an oral mucosa rather than an epidermal wound healing model, the effect of aPDT mediated by CUR-NP on the tongue mucosa was assessed. During the present study, the fungal infection increased the immuno-expression of both CKs evaluated, which might be attributed to the epithelial disarrangement and response to the fungal invasion. To the best of the authors’ knowledge, this was the first study that observed an increased expression of CKs as a result of OC. Other investigations reported alteration of CK expression as a result of oral lesions, such as lichen planus [54,55], and actinic cheilitis [56], but they did not associate CK expression with microbial biofilm. Kaminagakura et al, 2006 [57] evaluated the pattern of CK expression by IHC in biopsies of oral mucosa from patients with paracoccidioidomycosis showing pseudoepitheliomatous hyperplasia. In the oral mucosa of these patients, they found that CK1 and CK10 were not expressed in the hard palate, gingiva, and lips, and CK14 expression increased in the buccal mucosa and lips of oral infected mucosa [57]. Therefore, in accordance with this study, this investigation demonstrated that CK expression is altered in cases of oral infection.

Moreover, the present study showed that treatment with PS alone and nystatin also increased the immunolabelling for CK13 and CK14, similar to the untreated control group. As the treatment with PS alone did not reduce the fungal load, a high expression of both CKs observed in these groups was expected. However, although nystatin had reduced the fungal viability from tongues of mice, the expression of CK13 and CK14 was similar to the untreated control group, suggesting that nystatin was not able to improve the reorganization of the cytoskeleton of the epithelium even after seven days from the end of the treatment. Surprisingly, aPDT with free CUR resulted in reduced expression of both CKs. This was an unexpected outcome, as aPDT did not completely eliminate the fungal load from the tongue; therefore, CK expression was expected to be more intense, as observed in mice treated with nystatin. This result suggests that, concurrent with the reduction in the fungal viability, aPDT also contributed to the normal expression of CK13 and CK14 of the epithelial cells. However, the mechanism of such effects should be further investigated. Alternately, treatment with anionic and cationic CUR-NP (with and without light) resulted in expression of CK13 and CK14 similar to the infected mice. Concerning anionic CUR-NP, this result might be explained by the absence of antimicrobial activity. Although cationic CUR-NP had reduced the fungal viability, the increased expression of both CK might be justified by the CTAB in its composition, which showed in vitro cytotoxicity [31].

*C. albicans* is able to invade the epithelial layers and promote tissue damage by necrosis or apoptosis [5]. aPDT mediated by silicon phthalocyanine Pc4 promoted nuclear alterations in *C. albicans* similar to apoptosis, such as externalization of phosphatidylserine, chromatin condensation, and DNA fragmentation [58]. The reactive oxygen species produced by anticancer photodynamic therapy kill neoplastic cells by necrosis, apoptosis, or autophagy [59]. Therefore, in the present investigation, the TUNEL assay was performed in order to evaluate the safety of the consecutive applications of the aPDT on the hosts’ tissues. The findings showed that DNA damage was not observed after the treatments, especially after 24 h. An in vitro study, which also evaluated the safety of aPDT, observed no induction of apoptosis on human gingival fibroblasts and osteoblasts submitted to methylene blue at 50 μg/mL for 10 min followed by irradiation with a red diode laser at 12 J/cm^2^ [60]. During an ex vivo study, the TUNEL assay demonstrated that porcine skin samples inoculated with MRSA and submitted to aPDT (10 μM of porphyrin-based PS and 13.7 J/cm^2^) showed a slight increase in dead cells within the epidermis (16.55–35.8% ± 5%) compared with non-treated samples (15.3% ± 5%) [61]. Some studies have reported that blue light alone has an antimicrobial effect without harming the host tissue [62,63]. Mice burns infected with *P. aeruginosa* and irradiated with blue light (415 ± 20 nm, 55.8 J/cm^2^) not only showed a reduction of bacterial burden, but also rescued mice from death, and no DNA damage in the host tissue was observed [62]. Irradiation with blue light (415 ± 20 nm) of burned skin of mice infected with multidrug-resistant *Acinetobacter baumannii* reduced the bacterial load (55.8 J/cm^2^) and resulted in no significant DNA damage (195 J/cm^2^) in the cells of the epidermis evaluated by the TUNEL assay [63].

The present investigation demonstrated that the number of cells showing DNA damage after seven days increased in groups with a high amount of fungi, but not in groups with a reduction of fungal viability (cationic CUR-NP with and without light, aPDT with free CUR, and nystatin), which might indicate that DNA damage is due to the persistence of *C. albicans* in the tissue. Although, in the authors’ previous in vitro study, the cationic CUR-NP was toxic to mammal cells [31], in the present investigation, this formulation was not able to harm the tissue, probably because of the low CTAB concentration (2%).

To respond to *Candida* infection, the host cells synthesize pro-inflammatory cytokines and chemokines that mediate immune protection against *Candida* [64]. An in vitro study demonstrated that human oral and vaginal epithelial cells co-cultured with *C. albicans* produced tumor necrosis factor α (TNF-α) and interleukin 1α (IL-1α), but not interleukin 6 (IL-6) [65]. A protective response against *C. albicans* has also been associated with T helper (Th) in 17 cells induced by interleukin 1β (IL-1β), IL-6, and transforming growth factor β (TGF-β) [66], more so than with the classical Th1 response [67]. Unfortunately, the authors could not demonstrate the genetic expression of the inflammatory cytokines after treatments of OC because of the loss of samples during RNA extraction and purification (see Supplementary Material). The authors tried to improve the method previously reported [20], after careful considerations with an expert in molecular biology, by including the evaluation of RNA integrity. However, the authors observed a low yield and degradation of RNA. Only in the last attempts, with the remaining samples pooled, could they obtain a minimum amount of RNA for cDNA synthesis. Considering these samples (from AC + L−, CC + L−, AC + L+, and CC + L+ groups), the authors did not observe any increase in the inflammatory cytokines evaluated (TNF-α, IL-1β, and IL-6), although the control groups (C − L− and healthy mice) were not included (Figure S1). Other in vivo studies observed reduction of cytokine expression after aPDT associated or not with scaling/root planning for treating periodontitis [68,69].

## 4. Materials and Methods

### 4.1. Synthesis and Characterization of CUR-NPs

#### 4.1.1. PS and Light Source

Free CUR and CUR-NPs were used as PSs in this study. A stock solution of free CUR (260 μM) was prepared in dimethyl sulfoxide (DMSO) 10% diluted in sterile milli-Q water [15,16,18,19]. A blue (≈ 455 nm) LED (LXHL-PR09, Luxeon III Emitter, Lumileds Lighting, San Jose, CA, USA) device was elaborated as a handpiece by the Physical Institute of São Carlos—University of São Paulo (USP). The light emission end of the device had a diameter of 5 mm and a power output of 75 mW.

#### 4.1.2. Encapsulation of CUR in NPs

CUR-NPs were synthesized by the nanoprecipitation method [32], characterized as previously reported [31], and submitted to a patent deposited on the National Institute for Industrial Property (INPI) (process number BR 10 2016 015631 9). To perform this, a stock solution of CUR 0.4% was prepared in acetone. During the encapsulation process, an organic phase containing PLA (kindly provided by Professor Valtencir Zucolotto from University of São Paulo) at 0.5% in acetone was mixed with the stock solution of CUR. Then, an aqueous phase containing DEX 1% (Sigma-Aldrich Brasil Ltda, São Paulo, SP, Brazil) in milli-Q water was added by dropping to the organic phase. This procedure was performed at room temperature (25 °C) under slow magnetic rotation (90–100 rpm). Afterward, the solution was maintained under magnetic stirring for 10 min. The NP solution was kept at room temperature for 24 h to evaporate the solvent. This procedure resulted in anionic NP. To synthesize cationic CUR-NP, after the 24 h of acetone evaporation, CTAB (Sigma-Aldrich Brasil Ltda) 2% in sterile water and NaCl 1.76% was added [70]. The maximum concentration of CUR encapsulated in NP was 260 μM. Anionic and cationic NPs without CUR were also synthesized by the same method as the controls.

#### 4.1.3. Physicochemical Properties

Nanometric properties of CUR-NPs were characterized by size, PDI, and Zeta potential (determination of the superficial charge) using a Zeta Sizer NANO ZS (Malvern Instruments Ltd., Malvern, UK) at 25 °C.

#### 4.1.4. Morphology Analysis

CUR-NPs solutions were diluted in sterile ultra-pure water (1:200) and dried in a vacuum desiccator for 48 h, protected from light. The samples were placed in conductive stubs for carbon coating in the Sputter Coater (SCD 050 BAL-TEC). Particle morphology was analyzed by a FEG-SEM (Jeol, JSM 7500 F). Size was measured by ImageJ software (1.51j, National Institutes of Health, Bethesda, MD, USA).

#### 4.1.5. Encapsulation Efficiency (EE)

Regarding EE, 5 mL of the CUR-NPs at 52 μM were centrifuged at 6000× g for 10 min. The absorbance of the supernatant (free portion) was determined in a UV-visible spectrophotometer (SQ 4802 UV-Visible Double Beam Spectrophotometer UNICO, United Products and Instruments Inc., Dayton, NJ, USA). The EE was calculated using the following formula: EE% = (PStotal − PSfree) × 100/PStotal [31,35].

#### 4.1.6. Absorption Spectrum and Photostability

The absorbance of each PS (free CUR, and cationic and anionic CUR-NPs) at a concentration of 80 μM was determined in a plate reader in the UV-visible spectrum of 300–800 nm (Fluostar Omega, BMG LabTech, Offenburg, Germany). The photostability of CUR-NPs was compared with free CUR. Photodegradation was monitored by absorption spectrum between the wavelengths of 300–800 nm of the samples after illumination. CUR and CUR-NPs were exposed to different illumination intervals of a LED light source (≈455 nm) of 36 mW/cm^2^. Sample absorption was determined before (0 min) and after each irradiation time (1, 2.5, 5, 10, and 20 min, corresponding to 2.16, 5.4, 10.8, 21.6, and 43.2 J/cm^2^, respectively). Blank samples corresponded to NP solutions, whose absorbance was read to verify the hypothesis of absorption of the excipient solution (PLA and DEX) [31].

#### 4.1.7. Release of CUR from NPs

Samples of cationic and anionic CUR-NPs at 130 μM were incubated at 37 °C under agitation (100 rpm). Every 2 h, 2 mL of the samples was centrifuged at 6000× *g* for 10 min to separate the NPs from the solution and then the pellets were diluted in acetone to solubilize the unreleased CUR, whose absorbance was determined in a UV-visible spectrophotometer. The total CUR released was calculated by dividing the absorbance of each interval time by the maximum absorbance of the encapsulated CUR determined in the EE study [28,31].

### 4.2. In Vivo Study

#### 4.2.1. Animals

The authors used 235 female Swiss mice of 4–6 weeks of age, weighing approximately 20 g. The research protocol was approved by the Ethical Committee for Animal Research (“Comissão de Ética no Uso De Animais” CEUA; process 19/2015 approved on 8 December 2015) of the School of Dentistry, Araraquara, São Paulo State University (UNESP). The animals were kept in polypropylene cages with five animals in each cage, with controlled temperature (23 ± 2 °C) and humidity (55 ± 10%). The mice were maintained under a 12:12 light/dark cycle with light onset at 07:00 a.m. Standard chow (Guabi Rat Chow, Paulinia, SP, Brazil) and water were provided ad libitum.

#### 4.2.2. Oral Infection and Treatments

**Scheme 1:**
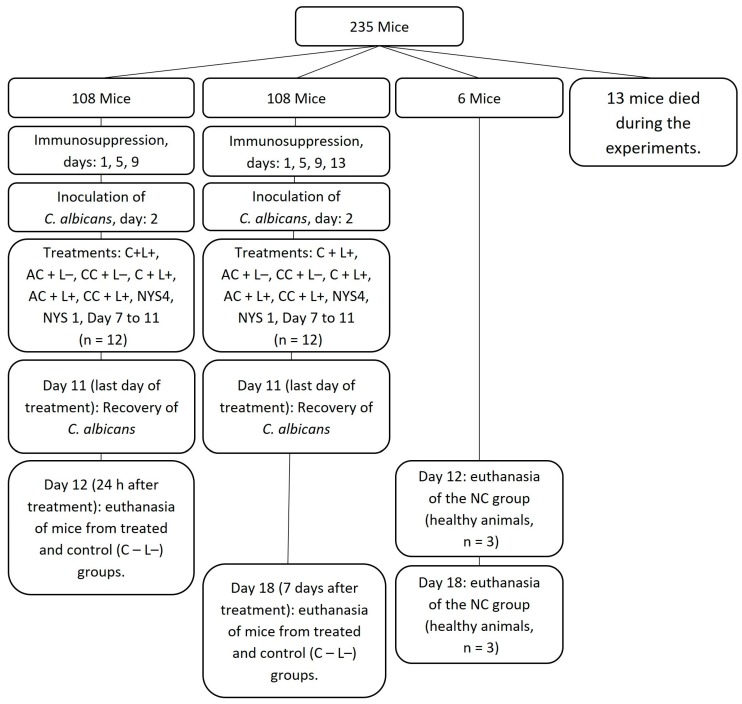
Study design. C + L+, AC + L+, and CC + L+ groups: animals treated with aPDT mediated by free CUR, and anionic or cationic CUR-NPs, respectively, and blue LED light; C + L−, AC + L−, and CC + L− groups: evaluation of each PS alone (free CUR, and anionic or cationic CUR-NP, respectively); NYS1 or NYS4 groups: animals treated with Nystatin 100,000 IU once (under anesthesia) or four times daily, respectively; C − L− (control) group: infected animals that did not receive any treatment; and negative control (NC) group: healthy and untreated. During the entire experimental period (18 days), tetracycline hydrochloride was given in drinking water.

A reference strain of *C. albicans* ATCC 90028 (Rockville, MD, USA) was kept in yeast nytrogen broth with 100 mM of glucose (YNBg) and glycerol 50% at −80 °C. During each experiment, the strain was thawed and grown in sabouraud dextrose agar with 5 mg/mL of chloramphenicol (SDAc), which was incubated at 37 °C for 48 h. Colonies were resuspended in YNBg and incubated at 37 °C for 16 h. This suspension was then diluted 1:20 in a fresh YNBg and incubated at 37 °C until the mid-log phase of growth (OD_540_: 0.548 ± 0.048), whose concentration was standardized at 5.8 × 10^6^ ± 4.2 × 10^6^ (mean ± standard deviation) CFU/mL. To induce the OC in animals, the methodology of Takakura et al., 2003 [71] was used with the modifications of Carmello et al., 2016 [20] to prolong the period of infection and to perform successive aPDT applications (Scheme 1). The animals were immunosuppressed with subcutaneous injections of prednisolone at the dose of 100 mg/kg on days 1, 5, 9, and 13. Tetracycline hydrochloride at 0.83 mg/mL was provided to the animals in the drinking water during the entire period. Oral inoculation of *C. albicans* was performed on day 2 by scrubbing a cotton swab immerged in the yeast suspension in the entire oral cavity. To do this, animals were anesthetized by an intramuscular injection of 50 μL of chlorpromazine hydrochloride at 2 mg/mL in each femur.

Treatments were performed daily from days 7 to 11. The groups PS only and aPDT involved free CUR (C + L− and C + L+, respectively), anionic CUR-NPs (AC + L− and AC + L+, respectively), and cationic CUR-NP (CC + L− and CC + L+, respectively). Each experiment was performed with 16–20 mice divided into four different groups (control, PS only, aPDT, and nystatin). Animals were allocated to the following groups: for aPDT, animals were anesthetized with an intraperitoneal injection of ketamine hydrochloride (100 mg/kg of body weight) associated with xylazine hydrochloride (10 mg/kg of body weight). Then, 50 μL of the PS at 260 μM was pipetted on the dorsum of the tongue. Following 20 min in the dark (pre-irradiation time), the tongue of the animals was illuminated for 7 min at 37.5 J/cm^2^ (C + L+, AC + L+, and CC + L+ groups). To verify a possible toxic effect of the applied PS, additional animals were treated only with the PS during the pre-irradiation and illumination times (C + L−, AC + L+, and CC + L+ groups). The control group (C − L−) received only sterile phosphate-buffered saline (PBS) during the same pre-irradiation and illumination times. The effect of light alone was not evaluated, as previous studies demonstrated no fungicidal effect of blue light at 37.5 J/cm^2^ [19,72]. To compare, two additional groups received topical application of nystatin 100,000 IU. Regarding the NYS1 group, nystatin was applied once a day for five consecutive days under anesthesia. To simulate the clinical use of nystatin, another group received nystatin for four times a day (intervals of four hours) during five consecutive days without anesthesia (NYS4). Moreover, in the NC group, animals were not inoculated with the fungi and did not receive any treatment. Twelve animals from each group were evaluated in each period of euthanasia [20], which was performed 24 h and 7 days after the last day of treatment for each group, with the exception of the NC group (*n* = 3).

Subsequent to the last fifth day of each treatment, *C. albicans* was recovered from the tongue of each animal by swabbing a mini cotton swab for 1 min on the dorsum of the tongue. The mini-swabs were kept in 1 mL of sterile saline solution, which was vortexed and serially diluted. Aliquots of 25 μL were spread in duplicate in SDA plates, which were incubated at 37 °C for 48 h for colony quantification (CFU/mL). No fungal recovery was performed seven days after the treatment because a previous study reported no significant difference between the log_10_ (CFU/mL) of *C. albicans* recovered 24 h and seven days after aPDT/no treatment [20].

#### 4.2.3. Euthanasia

The animals were euthanized 24 h (on day 12) or seven days (on day 18) after the fifth application of each treatment by a lethal dose of ketamine hydrochloride by intramuscular injection into the femur region. Then, the tongue of each animal was surgically removed. The anterior third and posterior third were removed and the dorsal region was divided in the longitudinal plane. One half of the tissue was fixed in 4% formaldehyde for histological, immunohistochemical (IHC), and terminal deoxynucleotidyl transferase (TdT)-mediated deoxyuridine triphosphate (dUTP)-biotin nick end labeling (TUNEL, DNA fragmentation) analyses. The other half was stored in RNA LATER at −80 °C for quantitative reverse transcription-polymerase chain reaction (RT-qPCR) analyses (Supplementary material).

#### 4.2.4. Histological Analyses

Following fixation in 4% formaldehyde buffered at Hidrogenionic potential (pH) of 7.2 with 0.1 M sodium phosphate, the samples were processed for paraffin embedding. The sections (5-μm thick) were adhered to glass slides and submitted to the periodic acid-Schiff (PAS) histochemical method and counterstained with hematoxylin to be observed in light microscopy (BX-51, Olympus Corporation, Shinjuku, Tokyo, Japan). The morphological alterations were evaluated according to the presence of yeast and filamentous cells, the organization of the epithelial layer, and the presence of inflammatory cells in the connective tissue.

#### 4.2.5. Immunohistochemistry (IHC) for Cytokeratins (CKs)

The sections of the tongue adhered to silanized glass slides were deparaffinized for avidin-biotin complex detection of CK13 and CK14. Antigenic retrieval was performed by heating the sections in 10 mM sodium citrate buffer, pH = 6 (for CK14), or 10 mM of tris(hydroxymethyl) aminomethane-ethylenediaminetetraacetic acid (TRIS-EDTA, TE) buffer, pH = 9 (for CK13), in a microwave oven (98 °C for 10 min). The samples were washed and immersed in 4% H_2_O_2_ for 10 min to inactivate the endogenous peroxidase. Following washing, the samples were incubated in a solution containing 2% bovine serum albumin (BSA, Sigma-Aldrich)/1% sodium azide (Sigma-Aldrich)/1% Triton X-100 (Sigma-Aldrich) for 20 min at room temperature. Subsequently, the sections were incubated in a humidified chamber at 4 °C overnight with mouse monoclonal anti-CK13 antibody (ab16112, ABCAM^®^), or mouse monoclonal anti-CK14 antibody (ab7800, ABCAM^®^), both diluted 1:500. The next day, the samples were washed in PBS and incubated with the second antibodies [goat anti-mouse horseradish peroxidase (HRP)/dihydrochloride (DAB) (ab64259, ABCAM^®^)] for 20 min. Subsequently, the samples were washed and incubated in streptavidin for 20 min (ab64259, ABCAM^®^). Next, the samples were marked by 4,6-diamino-2-phenylidole dihydrochloride (DAB; Invitrogen) and counterstained with Carazzi’s hematoxylin for analysis under light microscope (BX-51, Olympus Corporation, Shinjuku, Japan), and photographed at magnification 200× and 400×.

#### 4.2.6. Tissue Damage: Detection of Cell Death by the TUNEL Method

To evaluate the treatment safety, the TUNEL assay was performed using Click-iT^®^ TUNEL Alexa Fluor^®^ 594 Imaging Assay kit (Molecular Probes^®^), according to the manufacturer’s instructions. Briefly, sections were deparaffinized for a prolonged time, to remove paraffin as much as possible. Then, the samples were incubated with proteinase-K, a permeabilizing agent used to expose the DNA fragments, and fixed with 4% formaldehyde. A positive control of the reaction was made by incubating a slide from negative control (NC) group in 1 unit/µL of TURBO DNAse (Ambion, AM2238). Afterward, all samples were incubated with terminal deoxynucleotidyl transferase (TdT) reaction mixture, washed with 3% BSA and 0.1% Triton X-100 in PBS, followed by Click-iT Plus TUNEL reaction cocktails to mark cells with DNA fragmentation. Slides were washed with 3% BSA in PBS for 5 min. The DNA intercalator 4,6-diamidino-2-phenylindole (DAPI, Molecular Probes, NucBlue^®^ Fixed cell stain ready Probes, R37606) was used to counterstain the nuclei. Finally, the slides were assembled with fluoromount (CAT # 17984-25, Electron Microscopy Sciences, Fort Washington, WA, USA) and visualized in a fluorescence microscopy (Leica DMI3000 B).

### 4.3. Statistical Analyses

The values of log_10_ (CFU/mL) recovery from tongues of mice were submitted for statistical analyses. Normality and homoscedasticity were evaluated by Shapiro-Wilk and Levene tests, respectively. As the assumption of normality was met, but the homogeneity of variance was not met, data were analyzed by analysis of variance (ANOVA)/Welch and post-hoc of Games–Howell. The significance level adopted was 5% (α = 0.05). All analyses were performed using SPSS software version 20.0 (SPSS Inc., Chicago, IL, USA).

To quantify the IHC immunolabelling, the software ImageJ (1.51j, National Institutes of Health, Bethesda, MD, USA) was used as previously described [73,74]. The colorful images (.tiff) at magnification 200× were converted to gray scale, and the blue channel of the red-green-blue (RGB) stack was selected to remove the background. Then, the threshold was manually adjusted to separate the positive-IHC immunolabelling from the negative-IHC ones, by comparing with the original colorful image. Afterward, the epithelium region was selected (basal and spinous cell layers), and the immunolabelled area was measured as a percentage. Sections of two animals from each group were analyzed. Therefore an inferential statistical analyze was not performed [only descriptive statistics (mean and standard deviation) were shown].

The histopathological and TUNEL analyses were performed qualitatively.

## 5. Conclusions

This study encapsulated CUR in NPs, which improved the water solubility of CUR. However, free CUR showed a better photodynamic effect than CUR-NP, and nystatin once a day in anesthetized animals showed the best antifungal effect. DNA damage of the host tissue was related to the fungal infection, and not to the treatments performed. Furthermore, aPDT with free CUR resulted in expression of CK13 and CK14 of the epithelium of the tongue close to that observed in healthy mice, an outcome not observed for nystatin. Further studies should be developed to improve the release of CUR from NP and its antimicrobial effect, and to better understand the expression of CKs of the infected tissue after aPDT.

## 6. Patents

Authors report a patent deposited in the National Institute for Industrial Property (INPI) in Brazil on 4 July 2016, process BR 10 2016 015631 9, entitled “Processo de Obtenção de Nanopartículas Poliméricas Aniônicas e Catiônicas de Curcumina, Nanopartículas Obtidas e Seus Usos”.

## Figures and Tables

**Table 1:** Mean values of size, poly-dispersion index (PDI), and zeta potential (± standard deviation) of NPs formulations with and without CUR determined in dynamic light scattering (DLS) in a period interval of 0–29 days after the synthesis (*n* = 12).

	Size (nm)	PDI	Zeta Potential (mV)
**Anionic CUR-NP**	225 (±60.38)	0.15 (± 0.06)	−30 (±12.58)
**Cationic CUR-NP**	248 (±86.39)	0.21 (± 0.09)	+35 (±7.23)
**Anionic NP**	193 (±17.95)	0.15 (± 0.05)	−32 (±11.86)
**Cationic NP**	186 (±14.97)	0.14 (± 0.04)	+33 (±2.69)

## Supplementary Materials

The following are available.
