# Supplementary material for: Antimicrobial Photodynamic Therapy Mediated by Curcumin-Loaded Polymeric Nanoparticles in a Murine Model of Oral Candidiasis
Source: Molecules. 2018 Aug 19;23(8):2075. doi: 10.3390/molecules23082075 (PMC6222858; doi:10.3390/molecules23082075)
Supplement: Supplementary file 1 [file molecules-23-02075-s001.pdf]

Supplementary Material

Genetic Expression of the Inflammatory Cytokines by Quantitative Reverse Transcription-Polymerase Chain Reaction (RT-qPCR)

The expression of inflammatory cytokines was evaluated as previously described [20]. After the storage of the samples in RNA LATER (Ambion), samples were transferred to vials containing 1 mL of TRIZOL (Ambion), which were manually ground or mechanically homogenized in a mini-BEADBATER-16 processor (Model 607; 3450 rpm; Biospec product) with 500 mg of glass spheres (0,1 mm diameter glass, Biospec product) by vibration during 8 cycles of 40 seconds with intervals of 20 seconds at 4 °C. An aliquot of 500 μL was transferred to another microtube containing 0.1 mL of chloroform, which was homogenized and incubated for 3 minutes at room temperature. Following, the samples were centrifuged at 12,000 rcf (Microcentrifuge Eppendorf model 5415R, Hamburg, Germany) for 15 minutes at 4 °C. Then, the aqueous phase containing RNA was transferred to a new tube followed by the addition of 100 μL of isopropanol and 10% of sodium acetate (Ambion) to promote the precipitation of the RNA. The samples were incubated at –80 °C overnight. After the incubation time, samples were centrifuged at 12,000 RCF for 30 minutes at 4 °C. The supernatant was removed, and 0.5 mL of ethanol 75% was added to the pellet. Samples were centrifuged at 7,500 rcf for 5 minutes at 4 °C, and the supernatant was removed again. This process of washing samples was repeated thrice, and in the last washing ethanol 99% was used. Then, the pellet of RNA was dried at room temperature for 45 minutes. The RNA was resuspended in 10 mL of ultra-pure water (Invitrogen). The total RNA extracted was purified and DNAse-treated using a column-based purification kit (Qiagen micro-elute KIT), following the manufacturer's instructions. A second DNAse treatment was performed with Turbo DNAse, and RNA was purified using the Qiagen RNeasy MinElute kit (Qiagen). To quantify the RNA of the samples, their absorbance was read at 260 nm, and the ratio of 260 nm/280 nm was also determined in order to evaluate the purity of the samples. The absorbance was read after extraction and purification steps (3 stages), using a NANODROP DS-11+ Spectophotometer (DeNovix). The RNA integrity was evaluated running RNA samples in 1% agarose gel electrophoresis.

For each sample of total RNA, the cDNA was synthetized using the High Capacity DNA reverse Transcriptase KIT (Applied Byosystem), according to recommendations of the manufacturer. The cDNA was stored at –20 °C for posterior quantitative reverse transcription-polymerase chain reaction (RT-qPCR). After cDNA synthesis, the expression of the pro-inflammatory cytokines [interleukin 1β (IL-1β), interleukin 6 (IL-6), and tumor necrosis factor α (TNF-α)] involved in the inflammatory processes were assessed by each specific primer (TaqmanAssay - Applied Biosystems): βActin (control gene) = Mm00607939_S1; TNFα = Mm00443260_G1; IL-1β = Mm00434228_M1; IL-6 = Mm00446190_M1.

For the RT-qPCR reaction, 1 μL of the cDNA sample was added in 14 μL of the Taqman mix reaction. This mix consisted of 6.25 μL of the Taqman Assay kit (Applied Biosystems), 0.75 μL of the primer specific to each gene, and 7 μL of ultra-pure water. The samples were pipetted into PCR plates and sealed with adhesive. All procedures were performed on PCR Workstation and the surfaces were treated with RNAse (Applied Biosystens). Amplification analyzes were performed on thermocycler (CFX96 touch^{tm} real time PCR detection system, BioRad) and the amplification cycles were analyzed in the software BioRad CFX manager (BioRad). The thermal cycling consisted of 40 cycles of 2 min at 50 °C, 2 min at 95 °C for reverse transcription, 15 sec at 95 °C, 30 sec at 57 °C and 30 sec at 60 °C. In all experiments, negative controls (samples without primers) were performed to exclude possibilities of RNA or genomic DNA contamination

Individual samples manually ground showed low yield of genetic material (84.73% of genetic material was lost after purification procedures), hindering the cDNA synthesis. Therefore, samples were pooled according to their groups and mechanically homogenized, which increased the genetic material yielded after purification (56.11% of genetic material was lost). However, electrophoresis demonstrated degradation of RNA. Another attempt was performed with the remaining samples, which were pooled according to their groups (AC + L−, CC + L−, AC + L+, and CC + L+) and manually ground. Although a low yield was observed after purification (84.88% of genetic material was lost), electrophoresis demonstrated no degradation of RNA, and it was possible to standardize the samples in 1 μg/μL for cDNA synthesis. After qPCR, expression of

inflammatory cytokines was normalized by the expression of βActin gene. Each cytokine evaluated (TNF-α, IL-1β, and IL-6) showed similar expression among the groups (Figure 1S). Unfortunately, the samples of the other groups (C − L−, C + L−, C + L+, NYS1, and NYS4) were lost during the attempts to extract the RNA as described.

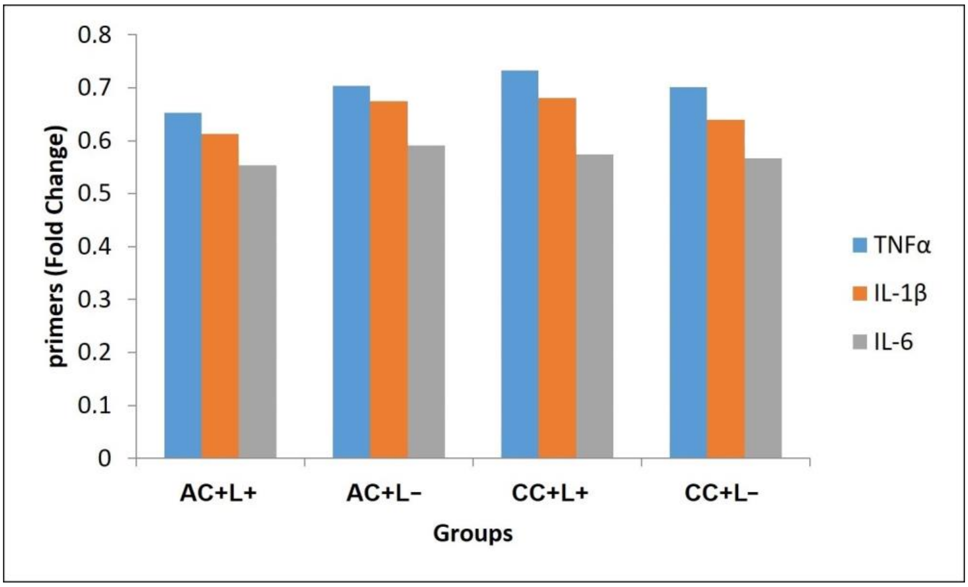

Figure S1. Mean values of gene expression for pooled samples normalized by the β-actin gene.
